# 9-(2-Chloro­benzyl­idene)anthracen-10(9*H*)-one

**DOI:** 10.1107/S160053681300809X

**Published:** 2013-04-05

**Authors:** Abdulrahman I. Almansour, Natarajan Arumugam, Raju Suresh Kumar, P. Vijayalakshmi, J. Suresh

**Affiliations:** a Department of Chemistry, College of Sciences, King Saud University, PO Box 2455, Riyadh 11451, Saudi Arabia; b Department of Physics, The Madura College, Madurai 625 011, India

## Abstract

In the title compound, C_21_H_13_ClO, the central anthracene system is distorted towards a boat conformation and the outer rings are not coplanar with the central ring [dihedral angles = 7.79 (1) and 11.90 (1)°]. The crystal structure features inversion dimers with graph-set motif *R*_2_^2^(18) formed by C—H⋯O inter­actions.

## Related literature

For ring conformations, see: Cremer & Pople (1975 ▶). For anthracene derivatives see: Alston *et al.* (1979 ▶); Kaplan & Conroy (1963 ▶); Meek *et al.* (1960 ▶); Singh & Ningombom (2010 ▶); Verma & Singh (1977 ▶). For hydrogen bonding, see: Bernstein *et al.* (1995 ▶).
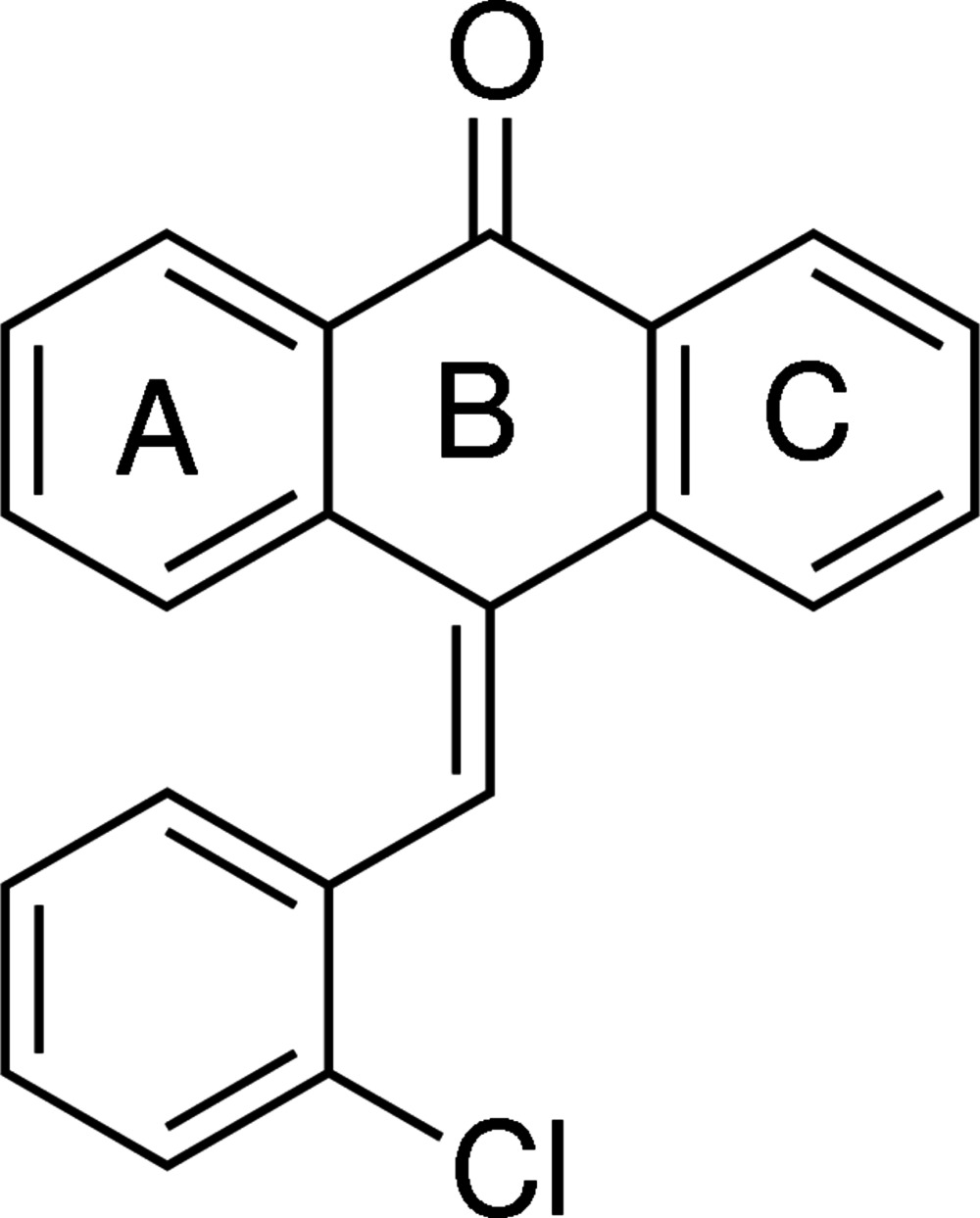


## Experimental

### 

#### Crystal data

C_21_H_13_ClO
*M* *_r_* = 316.76
Triclinic, 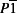

*a* = 7.9106 (10) Å
*b* = 8.3598 (10) Å
*c* = 12.6906 (15) Å
α = 82.813 (7)°
β = 83.979 (7)°
γ = 67.741 (6)°
*V* = 769.09 (16) Å^3^
*Z* = 2
Mo *K*α radiation
μ = 0.25 mm^−1^
*T* = 293 K
0.21 × 0.19 × 0.17 mm

#### Data collection

Bruker Kappa APEXII diffractometer
Absorption correction: multi-scan (*SADABS*; Sheldrick, 1996 ▶) *T*_min_ = 0.967, *T*_max_ = 0.974
11698 measured reflections
3182 independent reflections
2715 reflections with *I* > 2σ(*I*)
*R*_int_ = 0.034

#### Refinement

*R*[*F* ^2^ > 2σ(*F* ^2^)] = 0.045
*wR*(*F* ^2^) = 0.130
*S* = 1.06
3182 reflections
208 parameters
H-atom parameters constrained
Δρ_max_ = 0.34 e Å^−3^
Δρ_min_ = −0.50 e Å^−3^

### 

Data collection: *APEX2* (Bruker, 2004 ▶); cell refinement: *SAINT* (Bruker, 2004 ▶); data reduction: *SAINT*; program(s) used to solve structure: *SHELXS97* (Sheldrick, 2008 ▶); program(s) used to refine structure: *SHELXL97* (Sheldrick, 2008 ▶); molecular graphics: *PLATON* (Spek, 2009 ▶); software used to prepare material for publication: *SHELXL97*.

## Supplementary Material

Crystal structure: contains datablock(s) global, I. DOI: 10.1107/S160053681300809X/kj2220sup1.cif

Structure factors: contains datablock(s) I. DOI: 10.1107/S160053681300809X/kj2220Isup2.hkl

Supplementary material file. DOI: 10.1107/S160053681300809X/kj2220Isup3.cml

Additional supplementary materials:  crystallographic information; 3D view; checkCIF report

## Figures and Tables

**Table 1:** Hydrogen-bond geometry (Å, °)

*D*—H⋯*A*	*D*—H	H⋯*A*	*D*⋯*A*	*D*—H⋯*A*
C17—H17⋯O1^i^	0.93	2.60	3.482 (2)	159

Symmetry code: (i) 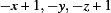.

**Fig. 1.**
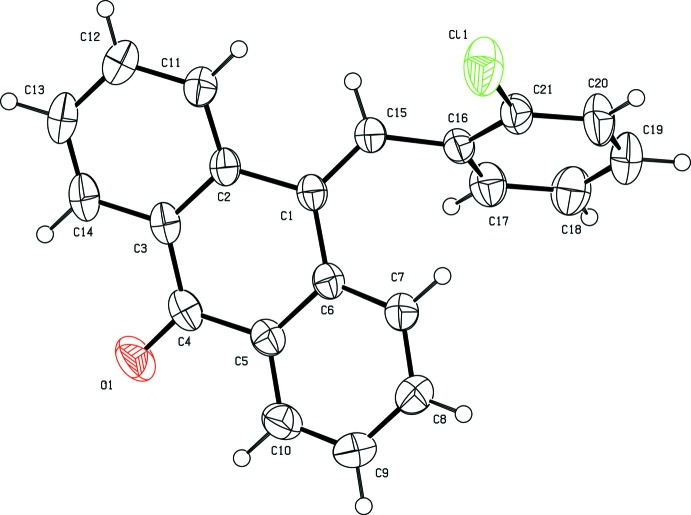
The molecular structure of the title compound, showing 40% probability displacement ellipsoids and the atom-numbering scheme.

**Fig. 2.**
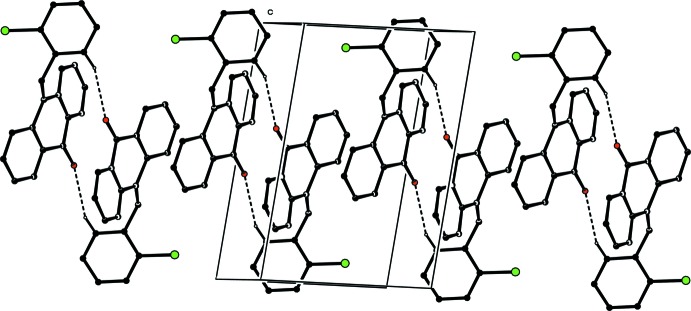
The partial packing diagram showing C—H···.O hydrogen bonding generating graph set motif R22(18).

## supplementary crystallographic information

### Comment

The compound anthracene has been known for a long time and its properties have been extensively studied. The regio and sterio-selectivity of substituted anthracenes in Diels-Alder reactions have been investigated and reported (Alston *et al.*, (1979); Meek *et al.*, (1960); Kaplan & Conroy, 1963; Verma & Singh, 1977; Singh & Ningombom, 2010). In view of this we have synthesized the title compound to study its crystal structure.

In the title compound (Fig 1),C_21_H_13_ClO, the benzene rings A and C in the anthracene moiety are almost individually planar with r.m.s deviation of 0.0071, and 0.0107 Å, respectively.The central anthracene ring B is distorted towards a boat conformation as evidenced by the puckering parameters q_2_ = 0.2074 (17) Å, θ = 76.8 (5)°, φ =5.9 (5)°(Cremer & Pople, 1975). The aromatic ring B is not coplanar with the aromatic rings A and C, as evidenced by the dihedral angles of 7.79 (1)° (A/B) and 11.90 (1)° (C/B) between them. The dihedral angle between the chlorophenyl ring and anthracene group is 55.69 (1)°. The carbonyl bond length C4=O1 [1.224 (2) Å] is somewhat longer than normal values due to involvement in a C—H···O contact. The twist of the chlorobenzene ring is indicated by the torsion angle C1—C15—C16—C17 is 60.72 (1)°. The range of C—C distances [1.365 (14)–1.484 (13) Å] and internal angles [117.00 (8)–121.55 (9)°] in the anthracene fragment are as expected for this type of molecule. In the crystal structure the C17—H17···O1 hydrogen bond connects two centrosymmetrically related molecules into dimers (Fig. 2) and generates a graph set motif of *R*_2_^2^(18) (Bernstein *et al.*, 1995). These centrosymmetric dimers are packed by weak Van der Waals interactions.

### Experimental

A mixture of anthrone (500 mg, 2.57) and 2-chlorobenzaldehyde (362 mg, 2.57 mmol) were dissolved in ethanol (10 ml) at room temperature. Then, the reaction mixture was saturated with gaseous hydrogen chloride for 1 h. The reaction mixture became dark and was thereafter heated to reflux for 1 h. After completion of the reaction as evidenced by TLC, the reaction mixture was cooled to room temperature. The solid product was filtered and dried at room temperature and recrystallized through ethyl acetate by slow evaporation technique. Melting point: 125°C,Yield: 85%

### Refinement

H atoms were placed at calculated positions and allowed to ride on their carrier atoms with C—H = 0.93 Å. *U*_iso_ = 1.2*U*_eq_(C) for CH_2_ and CH groups and *U*_iso_ = 1.5*U*_eq_(C) for CH_3_ group.

### Crystal data

**Table:**

C_21_H_13_ClO	*Z* = 2
*M**_r_* = 316.76	*F*(000) = 328
Triclinic, *P*1	*D*_x_ = 1.368 Mg m^−^^3^
Hall symbol: -P 1	Mo *K*α radiation, λ = 0.71073 Å
*a* = 7.9106 (10) Å	Cell parameters from 2000 reflections
*b* = 8.3598 (10) Å	θ = 1.6–26.5°
*c* = 12.6906 (15) Å	µ = 0.25 mm^−^^1^
α = 82.813 (7)°	*T* = 293 K
β = 83.979 (7)°	Block, colourless
γ = 67.741 (6)°	0.21 × 0.19 × 0.17 mm
*V* = 769.09 (16) Å^3^	

### Data collection

**Table:**

Bruker Kappa APEXII diffractometer	3182 independent reflections
Radiation source: fine-focus sealed tube	2715 reflections with *I* > 2σ(*I*)
Graphite monochromator	*R*_int_ = 0.034
Detector resolution: 0 pixels mm^-1^	θ_max_ = 26.5°, θ_min_ = 1.6°
ω and φ scans	*h* = −9→9
Absorption correction: multi-scan (*SADABS*; Sheldrick, 1996)	*k* = −10→10
*T*_min_ = 0.967, *T*_max_ = 0.974	*l* = −15→15
11698 measured reflections	

### Refinement

**Table:**

Refinement on *F*^2^	Primary atom site location: structure-invariant direct methods
Least-squares matrix: full	Secondary atom site location: difference Fourier map
*R*[*F*^2^ > 2σ(*F*^2^)] = 0.045	Hydrogen site location: inferred from neighbouring sites
*wR*(*F*^2^) = 0.130	H-atom parameters constrained
*S* = 1.06	*w* = 1/[σ^2^(*F*_o_^2^) + (0.0637*P*)^2^ + 0.2067*P*] where *P* = (*F*_o_^2^ + 2*F*_c_^2^)/3
3182 reflections	(Δ/σ)_max_ < 0.001
208 parameters	Δρ_max_ = 0.34 e Å^−^^3^
0 restraints	Δρ_min_ = −0.50 e Å^−^^3^

### Special details

**Table:**

Geometry. All e.s.d.'s (except the e.s.d. in the dihedral angle between two l.s. planes) are estimated using the full covariance matrix. The cell e.s.d.'s are taken into account individually in the estimation of e.s.d.'s in distances, angles and torsion angles; correlations between e.s.d.'s in cell parameters are only used when they are defined by crystal symmetry. An approximate (isotropic) treatment of cell e.s.d.'s is used for estimating e.s.d.'s involving l.s. planes.
Refinement. Refinement of *F*^2^ against ALL reflections. The weighted *R*-factor *wR* and goodness of fit *S* are based on *F*^2^, conventional *R*-factors *R* are based on *F*, with *F* set to zero for negative *F*^2^. The threshold expression of *F*^2^ > σ(*F*^2^) is used only for calculating *R*-factors(gt) *etc*. and is not relevant to the choice of reflections for refinement. *R*-factors based on *F*^2^ are statistically about twice as large as those based on *F*, and *R*- factors based on ALL data will be even larger.

### Fractional atomic coordinates and isotropic or equivalent isotropic displacement parameters (Å^2^)

**Table:**

	*x*	*y*	*z*	*U*_iso_*/*U*_eq_	
C1	0.3985 (2)	0.33688 (19)	0.34305 (11)	0.0387 (3)	
C2	0.3506 (2)	0.3882 (2)	0.45334 (11)	0.0399 (3)	
C3	0.4487 (2)	0.2788 (2)	0.53727 (12)	0.0442 (4)	
C4	0.6127 (2)	0.1233 (2)	0.51677 (13)	0.0477 (4)	
C5	0.6848 (2)	0.1024 (2)	0.40495 (13)	0.0442 (4)	
C6	0.5841 (2)	0.20832 (19)	0.32087 (11)	0.0400 (3)	
C7	0.6699 (2)	0.1932 (2)	0.21833 (13)	0.0489 (4)	
H7	0.6075	0.2639	0.1612	0.059*	
C8	0.8450 (3)	0.0755 (3)	0.20049 (16)	0.0617 (5)	
H8	0.8991	0.0676	0.1317	0.074*	
C9	0.9414 (3)	−0.0313 (3)	0.28405 (18)	0.0678 (5)	
H9	1.0591	−0.1116	0.2715	0.081*	
C10	0.8613 (3)	−0.0173 (2)	0.38533 (16)	0.0591 (5)	
H10	0.9256	−0.0885	0.4417	0.071*	
C11	0.2045 (2)	0.5395 (2)	0.47810 (13)	0.0486 (4)	
H11	0.1406	0.6159	0.4233	0.058*	
C12	0.1527 (3)	0.5783 (3)	0.58185 (14)	0.0563 (4)	
H12	0.0548	0.6797	0.5965	0.068*	
C13	0.2466 (3)	0.4659 (3)	0.66409 (14)	0.0624 (5)	
H13	0.2100	0.4903	0.7343	0.075*	
C14	0.3930 (3)	0.3193 (3)	0.64233 (13)	0.0578 (5)	
H14	0.4567	0.2452	0.6980	0.069*	
C15	0.2700 (2)	0.4023 (2)	0.27142 (12)	0.0440 (3)	
H15	0.1656	0.4945	0.2917	0.053*	
C16	0.2721 (2)	0.3476 (2)	0.16491 (11)	0.0431 (3)	
C17	0.2847 (3)	0.1809 (2)	0.15224 (15)	0.0578 (4)	
H17	0.2986	0.1002	0.2114	0.069*	
C18	0.2771 (3)	0.1331 (3)	0.05306 (17)	0.0674 (5)	
H18	0.2867	0.0206	0.0459	0.081*	
C19	0.2553 (3)	0.2514 (3)	−0.03530 (15)	0.0715 (6)	
H19	0.2496	0.2190	−0.1019	0.086*	
C20	0.2419 (3)	0.4165 (3)	−0.02466 (14)	0.0701 (6)	
H20	0.2275	0.4967	−0.0841	0.084*	
C21	0.2498 (2)	0.4635 (2)	0.07414 (13)	0.0515 (4)	
O1	0.6920 (2)	0.02023 (19)	0.58940 (10)	0.0694 (4)	
Cl1	0.23753 (11)	0.67325 (7)	0.08509 (4)	0.0865 (2)	

### Atomic displacement parameters (Å^2^)

**Table:**

	*U*^11^	*U*^22^	*U*^33^	*U*^12^	*U*^13^	*U*^23^
C1	0.0462 (8)	0.0407 (7)	0.0310 (7)	−0.0192 (6)	−0.0033 (5)	0.0010 (5)
C2	0.0480 (8)	0.0445 (8)	0.0323 (7)	−0.0236 (6)	−0.0025 (6)	−0.0012 (6)
C3	0.0555 (9)	0.0536 (9)	0.0324 (7)	−0.0306 (7)	−0.0065 (6)	0.0004 (6)
C4	0.0573 (9)	0.0510 (9)	0.0410 (8)	−0.0276 (7)	−0.0145 (7)	0.0071 (7)
C5	0.0498 (8)	0.0412 (8)	0.0442 (8)	−0.0190 (7)	−0.0101 (6)	−0.0006 (6)
C6	0.0455 (8)	0.0407 (7)	0.0373 (7)	−0.0194 (6)	−0.0062 (6)	−0.0023 (6)
C7	0.0496 (9)	0.0557 (9)	0.0398 (8)	−0.0176 (7)	−0.0030 (7)	−0.0050 (7)
C8	0.0530 (10)	0.0730 (12)	0.0532 (10)	−0.0154 (9)	0.0034 (8)	−0.0159 (9)
C9	0.0526 (10)	0.0647 (12)	0.0711 (13)	−0.0014 (9)	−0.0041 (9)	−0.0170 (10)
C10	0.0585 (10)	0.0490 (9)	0.0618 (11)	−0.0077 (8)	−0.0181 (8)	−0.0030 (8)
C11	0.0580 (9)	0.0502 (9)	0.0390 (8)	−0.0215 (7)	−0.0008 (7)	−0.0058 (7)
C12	0.0675 (11)	0.0596 (10)	0.0480 (9)	−0.0302 (9)	0.0096 (8)	−0.0173 (8)
C13	0.0845 (13)	0.0812 (13)	0.0338 (8)	−0.0438 (11)	0.0054 (8)	−0.0156 (8)
C14	0.0752 (12)	0.0744 (12)	0.0324 (8)	−0.0379 (10)	−0.0078 (7)	0.0007 (8)
C15	0.0452 (8)	0.0489 (8)	0.0331 (7)	−0.0127 (7)	−0.0021 (6)	−0.0018 (6)
C16	0.0406 (7)	0.0534 (9)	0.0336 (7)	−0.0155 (7)	−0.0049 (6)	−0.0015 (6)
C17	0.0709 (11)	0.0588 (10)	0.0472 (9)	−0.0288 (9)	−0.0075 (8)	0.0008 (8)
C18	0.0833 (13)	0.0667 (12)	0.0627 (12)	−0.0366 (11)	−0.0037 (10)	−0.0165 (10)
C19	0.0924 (15)	0.0898 (15)	0.0429 (10)	−0.0415 (12)	−0.0053 (9)	−0.0186 (10)
C20	0.1010 (16)	0.0799 (14)	0.0327 (8)	−0.0377 (12)	−0.0100 (9)	0.0008 (8)
C21	0.0621 (10)	0.0547 (9)	0.0353 (8)	−0.0193 (8)	−0.0060 (7)	−0.0008 (7)
O1	0.0783 (9)	0.0725 (9)	0.0492 (7)	−0.0220 (7)	−0.0208 (6)	0.0190 (6)
Cl1	0.1501 (6)	0.0594 (3)	0.0527 (3)	−0.0429 (3)	−0.0109 (3)	0.0024 (2)

### Geometric parameters (Å, º)

**Table:**

C1—C15	1.344 (2)	C11—H11	0.9300
C1—C6	1.477 (2)	C12—C13	1.383 (3)
C1—C2	1.484 (2)	C12—H12	0.9300
C2—C11	1.396 (2)	C13—C14	1.365 (3)
C2—C3	1.402 (2)	C13—H13	0.9300
C3—C14	1.401 (2)	C14—H14	0.9300
C3—C4	1.475 (2)	C15—C16	1.477 (2)
C4—O1	1.2243 (19)	C15—H15	0.9300
C4—C5	1.479 (2)	C16—C17	1.387 (2)
C5—C10	1.394 (2)	C16—C21	1.390 (2)
C5—C6	1.402 (2)	C17—C18	1.381 (3)
C6—C7	1.400 (2)	C17—H17	0.9300
C7—C8	1.377 (2)	C18—C19	1.378 (3)
C7—H7	0.9300	C18—H18	0.9300
C8—C9	1.386 (3)	C19—C20	1.366 (3)
C8—H8	0.9300	C19—H19	0.9300
C9—C10	1.370 (3)	C20—C21	1.374 (2)
C9—H9	0.9300	C20—H20	0.9300
C10—H10	0.9300	C21—Cl1	1.7412 (19)
C11—C12	1.378 (2)		
			
C15—C1—C6	124.09 (13)	C2—C11—H11	119.2
C15—C1—C2	118.82 (14)	C11—C12—C13	119.81 (17)
C6—C1—C2	117.00 (12)	C11—C12—H12	120.1
C11—C2—C3	117.96 (14)	C13—C12—H12	120.1
C11—C2—C1	122.31 (13)	C14—C13—C12	120.02 (16)
C3—C2—C1	119.67 (14)	C14—C13—H13	120.0
C14—C3—C2	119.68 (16)	C12—C13—H13	120.0
C14—C3—C4	119.23 (15)	C13—C14—C3	120.89 (16)
C2—C3—C4	121.07 (14)	C13—C14—H14	119.6
O1—C4—C3	121.67 (16)	C3—C14—H14	119.6
O1—C4—C5	121.00 (16)	C1—C15—C16	128.78 (14)
C3—C4—C5	117.22 (13)	C1—C15—H15	115.6
C10—C5—C6	120.50 (16)	C16—C15—H15	115.6
C10—C5—C4	118.28 (14)	C17—C16—C21	117.18 (15)
C6—C5—C4	121.07 (14)	C17—C16—C15	121.17 (14)
C7—C6—C5	117.52 (14)	C21—C16—C15	121.52 (15)
C7—C6—C1	122.48 (13)	C18—C17—C16	120.94 (17)
C5—C6—C1	119.91 (13)	C18—C17—H17	119.5
C8—C7—C6	121.19 (16)	C16—C17—H17	119.5
C8—C7—H7	119.4	C19—C18—C17	120.31 (19)
C6—C7—H7	119.4	C19—C18—H18	119.8
C7—C8—C9	120.66 (17)	C17—C18—H18	119.8
C7—C8—H8	119.7	C20—C19—C18	119.76 (17)
C9—C8—H8	119.7	C20—C19—H19	120.1
C10—C9—C8	119.24 (17)	C18—C19—H19	120.1
C10—C9—H9	120.4	C19—C20—C21	119.77 (18)
C8—C9—H9	120.4	C19—C20—H20	120.1
C9—C10—C5	120.85 (17)	C21—C20—H20	120.1
C9—C10—H10	119.6	C20—C21—C16	122.03 (17)
C5—C10—H10	119.6	C20—C21—Cl1	118.85 (14)
C12—C11—C2	121.55 (16)	C16—C21—Cl1	119.10 (13)
C12—C11—H11	119.2		
			
C15—C1—C2—C11	−21.6 (2)	C7—C8—C9—C10	−0.8 (3)
C6—C1—C2—C11	161.60 (14)	C8—C9—C10—C5	0.1 (3)
C15—C1—C2—C3	155.48 (15)	C6—C5—C10—C9	1.4 (3)
C6—C1—C2—C3	−21.3 (2)	C4—C5—C10—C9	−174.18 (17)
C11—C2—C3—C14	3.0 (2)	C3—C2—C11—C12	−2.4 (2)
C1—C2—C3—C14	−174.19 (14)	C1—C2—C11—C12	174.72 (15)
C11—C2—C3—C4	−175.59 (14)	C2—C11—C12—C13	0.1 (3)
C1—C2—C3—C4	7.2 (2)	C11—C12—C13—C14	1.6 (3)
C14—C3—C4—O1	6.6 (2)	C12—C13—C14—C3	−1.0 (3)
C2—C3—C4—O1	−174.78 (15)	C2—C3—C14—C13	−1.4 (3)
C14—C3—C4—C5	−169.63 (14)	C4—C3—C14—C13	177.23 (16)
C2—C3—C4—C5	9.0 (2)	C6—C1—C15—C16	9.2 (3)
O1—C4—C5—C10	−11.7 (2)	C2—C1—C15—C16	−167.39 (15)
C3—C4—C5—C10	164.58 (15)	C1—C15—C16—C17	60.7 (2)
O1—C4—C5—C6	172.76 (15)	C1—C15—C16—C21	−123.51 (19)
C3—C4—C5—C6	−11.0 (2)	C21—C16—C17—C18	0.6 (3)
C10—C5—C6—C7	−2.2 (2)	C15—C16—C17—C18	176.60 (17)
C4—C5—C6—C7	173.23 (14)	C16—C17—C18—C19	−0.5 (3)
C10—C5—C6—C1	−178.83 (15)	C17—C18—C19—C20	0.3 (3)
C4—C5—C6—C1	−3.4 (2)	C18—C19—C20—C21	−0.2 (3)
C15—C1—C6—C7	26.3 (2)	C19—C20—C21—C16	0.3 (3)
C2—C1—C6—C7	−157.05 (14)	C19—C20—C21—Cl1	178.61 (17)
C15—C1—C6—C5	−157.24 (15)	C17—C16—C21—C20	−0.5 (3)
C2—C1—C6—C5	19.4 (2)	C15—C16—C21—C20	−176.50 (17)
C5—C6—C7—C8	1.6 (2)	C17—C16—C21—Cl1	−178.81 (13)
C1—C6—C7—C8	178.12 (16)	C15—C16—C21—Cl1	5.2 (2)
C6—C7—C8—C9	−0.1 (3)		

### Hydrogen-bond geometry (Å, º)

**Table:**

*D*—H···*A*	*D*—H	H···*A*	*D*···*A*	*D*—H···*A*
C17—H17···O1^i^	0.93	2.60	3.482 (2)	159

Symmetry code: (i) −*x*+1, −*y*, −*z*+1.
